# The evolution of speech communication devices for anarthria: a review

**DOI:** 10.1007/s00415-026-13734-z

**Published:** 2026-03-13

**Authors:** Catherine T. Jones, Erik R. Hill

**Affiliations:** Department of Biological Sciences, Seton Hall University, 220 McNulty Hall, 400 South Orange Ave, South Orange, NJ 07079 USA

**Keywords:** Anarthria, Brain-computer interface, Speech communication

## Abstract

Anarthria is a lack of verbal communication caused by physiological disturbances in the motor pathway. While affected individuals retain the ability to comprehend and produce speech, orofacial paralysis renders them unable to execute speech. Anarthria can be caused by amyotrophic lateral sclerosis, stroke, traumatic brain injury, and other etiologies that affect the descending motor pathway. A wide range of technologies has been developed and tested to improve communication efficiency for patients with anarthria and accompanying paralysis. This review evaluates three key eras of communication device development. First, before implantation devices gained traction, many communication devices revolved around blinks, head and eye tracking, and non-invasive brain recording. Second, implanted cortical neuroprosthetics were designed to improve accuracy and speed of communication. Finally, the review analyzes the future era, where accessibility, patient comfort, and broader applications of neural analysis elevate communication for patients with anarthria to match fluid communication. Restoring speech communication in patients with anarthria is vital to improve their quality of life. Therefore, understanding communication device efficiency and its future trajectory is of utmost clinical importance.

## Introduction

Anarthria is a lack of verbal expression caused often by a physiological disturbance or injury to the dominant hemisphere [1]. Anarthria onset comes rapidly in different etiologies, including amyotrophic lateral sclerosis (ALS) [2, 3] or ischemic injury [4, 5]. Individuals with anarthria and quadriplegia are adults who retain language skills including written, oral, and reading comprehension; however, they lack the motor function to enact these functions (ICD-10-CM Diagnosis Code R47.1). Anarthria has many impacts on quality of life, but the most imperative is a desire to communicate without being able to [6, 7].

**Fig. 1:**
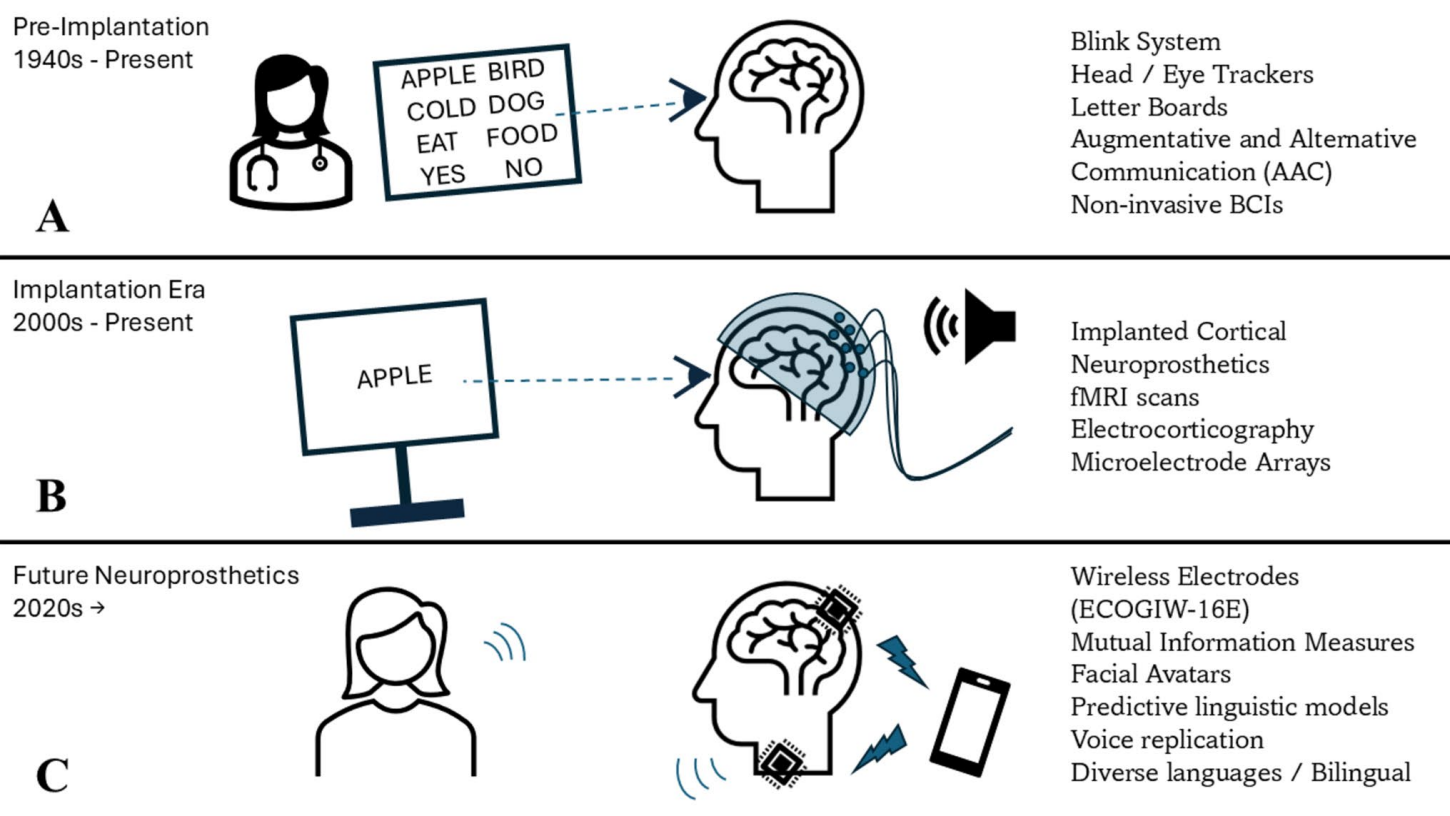
The three eras of speech communication devices for anarthria treatment. **A** Treatments before electrode implantation: blinking systems, head and eye trackers, letter boards, augmentative and alternative communication devices, and non-invasive BCIs such as electroencephalography. **B** Treatments involving implanting electrodes: implanted cortical neuroprosthetics, electrocorticography, and microelectrode arrays. **C** The future of speech communication devices: wireless electrodes, mutual information measures for enhanced neural recording, and nonverbal adaptations like facial avatars. Diagram was generated in PowerPoint 365 (RRID:SCR_023631)

The focus of this review is to discuss the major intervention strategies undertaken by clinicians to treat anarthria. These efforts fall into three general eras marked by improvements in technology (Fig. 1). The first era is a pre-implant era when the use of alternative communication efforts was the sole, favorable intervention. In the twenty-first century, cutting-edge developments in subcranial implants mark the current era of anarthria treatments. Finally, we discuss future efforts of novel decoding strategies on neural firing to understand how interference from non-speech-related functions relates to communication or to develop real-time decoding strategies of words that the participant generated from within their mind. While part of this review is segmented into non-implantable and implantable devices, both options are being concurrently researched as anarthria treatments.

## Pre-implant

The pre-implantation era started in the mid-twentieth century [8]. This era stretches into the present as research continues to explore avenues toward enhancing speech communication devices without implantation. In this era, speech rehabilitation for patients with anarthria was often confined to eye blink binary systems of communication, letter boards, and augmentative and alternative communication (AAC) devices like eye and head trackers [9]. In eye blink binary systems, the participant can only distinguish “yes” or “no” or identify a choice between options designated by the number of blinks required (Fig. 1A). This is most often the beginning form of communication for a recently developed anarthria patient [9]. Soon after, patients often progress to letter boards for communication, using their eyes to select letters and spell out their intended messages. For patients who are not paralyzed or exhibit severe paresis, keyboards and touchscreens provide written communication when vocalization is inhibited [10]. Touchscreen technology has developed to provide a more dynamic range of communication for these patients but requires limb mobilization for use [11]. However, for paralyzed or severely weakened patients, eye and head tracking devices have been used to improve communication. Although a step toward communication, this vehicle is often tedious for patients, as it takes a much longer time to meticulously spell out a message than it is to speak it and can be inaccessible to patients with more severe disabilities [12].

As interest in enhancing the efficacy of communication intervention increased, brain-computer interfaces (BCIs) were integrated. A BCI is a computer system that decodes a user’s neural activity and transmits that information to an attached device to perform the intended function [13, 14]. In speech communication, BCIs decode intended speech and operate with an external device to communicate effectively, either through selection, typing, or eventually text generation [15]. Electrooculograms have been used to non-invasively record electrical signals from eye movement for communication [10]. By placing electrodes around the eyes, a computer can interpret the eye movement for direction and blinks as selection tools. Using this technology, patients can select letters or words on their own and hands-free. There have also been speech-generating devices (SGD) used for communication that vocalize written speech [16]. The computer spoken text can be generated by various inputs such as typing or tapping from the user or with eye trackers that spell the desired communication; then the SGDs vocalize the message for more interactional communication [17]. An example of SGD communication is how Stephen Hawking communicated answers to pre-delivered questions during televised interviews [18].

Although the technological adaptations of eye-tracking devices have made them more accurate and sensitive, they are still only applicable in situations where lighting and postural conditions are ideal [19, 20]. A study was able to use a non-invasive BCI positioned above the parietal lobe to spell words indicated by a patient without needing head or eye tracking devices [21]. When provided a letter board, like patients using a head or eye tracker, the participant was able to identify and select letters without head or eye movements. Using electroencephalography on the skull, the participant’s cortical activity was read via event-related potentials (P300) and interpreted as letter selection. Thus, without tracking device dependency, the non-invasive BCI spelling method overcomes the idealistic nature of tracking devices. Still, these noninvasive BCI enhancements do not negate the tedious aspect of spelling words to communicate. One study tried to combine noninvasive BCI spelling models into a hybrid, attempting to make spelling communication more effective and less tedious for patients [22]. Although this was foundational work for BCI spelling technology, the technology did not spell fast enough, especially for fluid communication efficiency. When discussing the importance of fluid communication efficiency, this is predominantly characterized by the rates of interpretation and whether it mimics the flow of conversations carried out by speakers who do not utilize communication devices [23, 24]. Therefore, research into implanted BCIs for faster, holistic communication was necessary.

## Implant

Once the implantation of BCIs was growing traction in the 2000 s, they did not immediately remediate the concern of tedious spelling. A study used implanted BCIs in tandem with an auditory event-related potential (P300) spelling system for patients with locked-in syndrome to communicate [22]. This system utilized a letter board on a 5 × 5 grid with corresponding numerical identifiers for the patient to spell out communication without muscular involvement. Another study used similar implantable BCIs to coordinate neural activity with a cursor click methodology of typing out messages [25]. The participant was able to type 27 correct characters a minute without movement, the fastest typing speed using a BCI. Within the same realm of spelling for communication, another study utilized a fully implanted subdural BCI to coordinate attempted hand movements with a typing program [26]. After 28 weeks, the patient with late-stage ALS was able to control a computer typing program with hand movement, averaging 2 letters a minute. A similar study utilized an intracortical BCI to interpret attempted handwriting gestures into computer generated text for communication [27]. The participant had paralysis due to a spinal cord injury and was able to type 90 words per minute using the interpretative BCI with 94.1% accuracy without autocorrect and 99% accuracy with general autocorrect. These versions of implantable BCIs have improved from head and eye trackers but continue to lack the speed and flexibility of spoken communication where speaking rates range from 120 to 150 words per minute [28].

Developing technology has revolutionized communication. Instead of relying on spelling, whole words can now be decoded and interpreted [29]. Implanted BCIs serve as a new form of AAC devices that could go beyond tracking control or spelling for more effective communication via synthesized speech [30]. The increase in speed that synthesized speech provides over tracking and spelling is imperative for enhancing communication technology to accurately reflect fluid speech and allow patients to engage in meaningful dialog [31]. Implanted BCIs culminate into neuroprosthetics to create this more fluid communication route. Neuroprosthetics use algorithms to translate neural activity recorded by BCIs during volitional speech into text, sounds, and facial movements [32]. This allows neuroprosthetics to decode the corresponding words, phrases, and sentences from brain activity and display them on screen or read them aloud for communication (Fig. 1B). They are also referred to as implanted cortical neuroprosthetics (ICNs), due to their invasiveness via surgical implantation, process of recording the brain’s cortex, and aim to replace neural pathways rather than rehabilitate [30]. Although these implanted BCIs have much higher spatiotemporal resolution, are more robust against interference concerns, and can target neural populations more specifically in comparison to their non-invasive counterparts, they are also high-risk, inflexible after implantation, and expensive [33].

A 2009 study tested the efficacy of a brain-machine interface using invasive implanted electrodes on the left precentral gyrus for reading cortical activity related to speech planning [34]. Although this technology could only decode vowel pronunciations rather than full words, it laid the groundwork for electrode implantation technology for recording, analyzing, and decoding neural activity as attempted speech. From the 2010 s to present day, studies have inquired into the ability to produce full words with more electrode implantations. Electrocorticography is the recording of neural activity by electrodes placed on the surface of the cerebral cortex and serves as the key for recording neural data in neuroprosthetics [35, 36]. Before application for patients with anarthria, foundational neuroprosthetic work was conducted on patients who could speak aloud to test accuracy and comparability [37]. Five participants already undergoing intracortical monitoring for epilepsy treatment tested the neuroprosthetic where the decoder was able to synthesize speech in both out loud speech and silent miming.

Alongside this work, neuroprosthetic testing of words and phrases was already being conducted on patients with anarthria, with improving accuracy over time. One study had a 25.6% word error rate and 47.1% word accuracy rate with a 50-word vocabulary over 22 h of cortical activity recording [35]. The decreased error rate was achieved by recording the neural activity and using a decoding algorithm that predicted the words from the vocabulary that would most likely match the neural pattern based on a previous training period. Although this was a grand step for neural decoding, the high error and low accuracy rates prompted additional refinement in neuroprosthetic technology. Another study used a three-step decoding model, rather than a purely predictive model [38]. It would decode the neural activity, verify the accuracy of the decoding, and then classify the waveforms as different words based on training data. This extra verification step increased the decoding accuracy, boasting classification accuracies of 87.7% and 98.5% for two stroke-caused anarthria participants [38]. The difference between these two patients was the number of electrodes and placement, where the second participant had significantly more electrodes that were less spaced out, a reason for their far higher accuracy rate. The study also tested interference concerns, where auditory and visual stimuli had the potential to misfire the neuroprosthetic, as in triggering the neuroprosthetic in absence of a speech attempt. This was to analyze the neuroprosthetics’ specificity regarding external stimuli in real-world settings. They found that because of the verification step in the decoding model, zero false positives, or misfires occurred during usage. Therefore, accurate and specific neuroprosthetic models are being developed currently that decode full speech attempts without the need for spelling or eye tracking.

In all previously mentioned studies, the neuroprosthetic has been trained and analyzed on prompted communication. Principally, the participant is shown words and then prompted to attempt to say them [34, 35, 37, 38]. However, a 2024 study has analyzed neuroprosthetic usage in free, unprompted communication [39]. The participant was implanted with microelectrode arrays instead of electrocorticography, meaning the electrodes were inserted into the brain tissue rather than sitting on the surface of the cortex. Additionally, instead of the three-step decoding model, this decoding system relied on language learning models to interpret neural activity as predicted phonemes, those phonemes into predicted words, and those words into an organized sentence [39]. Drawing from many phoneme decoding algorithms that have been proven to correctly classify phonemes from neural activity, this premise served as an effective foundation [40]. Despite lacking a verification step, the study achieved a 2.7% word error rate over 22,000 + sentences. In free conversation, the neuroprosthetic interpreted 52.9% of sentences 100% correct, 32.3% mostly correct, and 14.8% incorrect [39]. Although sentence prediction rates can be higher, this is one of the most accurate neuroprosthetics to date that has been tested on free conversation, meaning the largest vocabulary. It also incorporated SGDs to read the decoded sentence aloud in a voice similar to the participant before he developed anarthria due to amyotrophic lateral sclerosis. Other studies have similarly incorporated pre-paralysis vocal output mimicry to enhance the fluency and flow of communication [31].

Currently, invasive methods such as electrocorticography or microelectrode arrays have been the most accurate and specific speech decoding techniques developed [41]. Noninvasive methods rely on participants spelling their intended words either with an external BCI or eye tracker. Implanted electrodes allow for word and sentence decoding in real-time, providing a more efficient mechanism for communication that increases quality of life for patients who have lost such a vital social skill [29]. However, the invasiveness and permanence of implantation are major drawbacks for this technology for many patients, in both financial and personal barriers.

## Future

The future of neuroprosthetics for communication rehabilitation in patients with anarthria is diverse in both technological innovation and application. Currently, accurate neuroprosthetics rely on invasive measures of cortical reading with external pedestals and wires attached to the skull to connect with the computer. This carries drawbacks in comfort, mobility, esthetics, and accompanying maladies such as inflammation or glial scarring [42]. In 2014, the development of wireless electrocorticography arrays started, and their future application in BCI communication could serve as a solution to the esthetic, mobility, and comfort issues resultant from an external direct connection to a computer [43]. ECOGIW-16E are externally rechargeable wireless electrodes that have proven effective in freely moving primate studies for cortical reading and stimulation [44]. These will also establish a safer and more effective route for patient monitoring by neglecting the need for cables exiting the skull. Although wireless electrocorticography will improve mobility, it still requires invasive brain surgery for implantation (Fig. 1C). There are no non-invasive neural reading technologies available yet that are more precise and accurate than invasive methods [15]. However, for increased accessibility, research has been oriented to improve the accuracy and specificity of noninvasive BCIs such as electroencephalography, event related potential, motor evoked potential, error-related potential, and steady state visually evoked potential [45].

There has also been increasing research in improving the accuracy of neural recording and decoding through different cortical reading methodologies. One study developed ‘Mutual Information’ measures in contrast to electrocorticography [46]. This method decodes neural activity using linear and nonlinear dependencies and was found to detect early prefrontal and premotor activations before speech onset in comparison to the standard. Additionally, it was more sensitive to the spatiotemporal elements of speech activation and execution occurring within the brain, showing a more accurate method for neuroprosthetic decoding of cortical activity.

Present neuroprosthetics have been proven effective in accurately decoding words and sentences from participants but have not been tested for personalized aspects of speech such as intonation, cadence, and facial expressions [29]. Two studies were able to synthesize speech from neural recordings by computer generating a voice similar to the participant’s voice before paralysis to read the predicted sentence [29, 47]. Additionally, to reflect the importance of nonverbal communication, the study developed a facial-avatar brain-computer interface to decode neural activity related to speech gestures and create a virtual, moving avatar for the participant. With the direct goal of improving communication for a wide variety of patients, decoding words and sentences is not the upper limit. Human communication is a combination of words, non-verbal cues, and gestures. Creating an avatar that can reflect orofacial expressions is integral for neuroprosthetic designs to improve communication in every aspect for paralyzed patients [48].

When looking into the future of neuroprosthetics, the diversity of language and inclusion of non-Germanic alphabets should be explored as current neuroprosthetic developments are predominantly English centered. In 2025, a bilingual neuroprosthetic recorder was generated and tested on an individual fluent in English and Spanish [49]. The decoding models relied on the shared neural pathways across the two languages in the participant’s speech-motor cortex and could accurately interpret neural activity in English and Spanish without needing to create separate decoders for each specific language. Thus, the study provides insight into how current neuroprosthetic decoding models can be trained to incorporate different languages without needing to create new decoding infrastructure. However, this study only tested a participant who spoke English and a second language, Spanish, that also used the Latin alphabet. Therefore, scientific inquiries into tonal monosyllabic languages, including Mandarin Chinese, and other tonal languages, including Vietnamese, should be researched. In 2025, a Mandarin BCI was developed that could decode 394 syllables from neural activity, resulting in a 71.2% accuracy rate [50]. The researchers proved that neuroprosthetic technology can decode tonal and specific syllables; therefore, providing a foundation for future enhancement that surpasses phoneme restraints. This research field is imperative to ensure inclusion and accessibility of this technology across language differences.

Yet neuroprosthetic technology is not confined to communication rehabilitation. The premise of electrode implantation for cortical activity decoding has broad implications in the realm of robotic prosthetics [51], brain activity reading for comatose patients [52], and treatments for other causes of anarthria like multiple sclerosis, meningitis, and traumatic brain injuries [11]. Additionally, there is research testing the realm of virtual reality as BCIs for embodied rehabilitation [53]. The vast potential of this technology warrants further research into its accuracy, accessibility, and applications.

Communication is an integral aspect of human socialization, for patients who have lost that ability via anarthria, neuroprosthetics are their chance for reestablishing connections. Therefore, future studies are vital for the enhancement of this technology to provide a bridge of communication for all people, regardless of motor capabilities.

## Discussion

This review analyzed how communication technologies have evolved and continue to evolve for improving quality of life for patients with anarthria. Communication is vital for human socialization, and thus many patients who lose communication ability suffer from isolation and depression [54]. Therefore, analyzing improvement in communication efficiency and its trajectory is of utmost clinical importance.

Although current neuroprosthetics have been proving effective, future directions are focused on honing mobility, accessibility, and programming in communication technology. However, in the 30 years that Stephen Hawking used an SGD to speak, he came to identify the synthesizer sound as his own ‘voice’ [18]. This highlights that while research discoveries are closer to mimicking how vocalization is generated, this work should include the agency of what a person considers their ‘voice.’

Neuroprosthetics also have multiple applications beyond communication, where brain-computer interfaces are implemented in limb paralysis, stroke, and mental health rehabilitation as well as in tandem with virtual reality clinical care [55]. Improved neuroprosthetic technology has implications for restoring fluid communication for thousands of patients suffering from isolation due to communication inabilities caused by anarthria. Neuroprosthetics continue to expand our understanding of the functions and mechanisms of cortical activity and provide a strong foundation for future clinical interventions requiring instantaneous neural activity decoding.

## Conclusion

In this review, the evolution of speech communication devices was analyzed across non-implantable, implantable, and future treatments for patients with anarthria. Non-implantable methods of communication covered consist of binary blink systems, letter boards, and AAC devices. These allow communication avenues for anarthria patients but are limited in speed and ability with fully paralyzed patients. Implantable devices use BCIs to interpret cortical activity into words and sentences for more efficient communication. However, these continue to be limited by the invasive nature of implantation and accuracy. When addressing clinical decisions on cutting-edge technologies, the reality of anarthria and speech communication devices should be understood within the holistic context of the patient’s need. For some anarthria cases, implantation could be considered too invasive, expensive, or have limited patient value and therefore may not be a viable treatment option even if implantation leads to more accurate and efficient communication. Future neuroprosthetic accessibility will be improved by fewer mobility restraints, improving software, expanding languages used in models, and including communication features outside of direct word translation such as facial expressions. Continued technological efforts that expand communication options and improving their accessibility will be critical in providing optimal anarthria care.

## Data Availability

Data supporting the results reported in this article can be found using the reference list at the end of this manuscript. In gathering the data presented, the reviewers conducted a comprehensive literature review selecting primary research into anarthria, neuroprosthetics, and BCI technology. All studies were peer-reviewed and moved the needle in understanding the etymology of anarthria, speech communication devices, and future technological advancements within the review scope. Additionally, supplemental research articles were included to contextualize the evolution of speech communication devices and the impact anarthria has on patient quality of life. All data were obtained from online access to journals, websites, and textbooks.

## Abbreviations

AAC: Augmentative and alternative communication
ALS: Amyotrophic lateral sclerosis
BCI: Brain–computer interface
ICN: Implanted cortical neuroprosthetics
P300: Event-related potential
SGD: Speech-generating devices
